# Association Between Metabolic Syndrome and Mortality: Prospective Cohort Study

**DOI:** 10.2196/44073

**Published:** 2023-09-05

**Authors:** Wenzhen Li, Dajie Chen, Ying Peng, Zuxun Lu, Mei-Po Kwan, Lap Ah Tse

**Affiliations:** 1 Jockey Club School of Public Health and Primary Care The Chinese University of Hong Kong Hong Kong China (Hong Kong); 2 Department of Health Service and Management Wuhan Polytechnic University Wuhan China; 3 Department of Communicable Diseases Control and Prevention Wuhan Center for Disease Control and Prevention Wuhan China; 4 Department of Social Medicine and Health Management School of Public Health, Tongji Medical College Huazhong University of Science and Technology Wuhan China; 5 Department of Geography and Resource Management The Chinese University of Hong Kong Hong Kong China (Hong Kong); 6 Institute of Space and Earth Information Science The Chinese University of Hong Kong Hong Kong China (Hong Kong); 7 Institute of Future Cities The Chinese University of Hong Kong Hong Kong China (Hong Kong); 8 Shenzhen Municipal Key Laboratory for health Risk Analysis Shenzhen Research Institute of the Chinese University of Hong Kong Shenzhen China

**Keywords:** metabolic syndrome, mortality, heart disease, diabetes mellitus, cancer

## Abstract

**Background:**

Metabolic syndrome (MetS) is a common metabolic disorder that results from the increasing prevalence of obesity, which has been an increasing concern in recent years. Previous evidence indicated that MetS was associated with mortality; however, different definitions of MetS were used. In 2005, the National Cholesterol Education Program (NCEP) Adult Treatment Panel (ATP) III updated the definition of MetS, which has since been widely adopted. Therefore, it is necessary to conduct a novel study among other populations and countries with a larger sample size using the updated definition of MetS and death code to examine the association of MetS with all-cause and cause-specific mortality.

**Objective:**

We aimed to examine the associations of MetS with all-cause and cause-specific mortality.

**Methods:**

A total of 36,414 adults were included in this study, using data from the National Health and Nutrition Examination Survey (NHANES) III (1988-1994) and the continuous NHANES (1999-2014) in the United States. Death outcomes were ascertained by linkage to National Death Index records through December 31, 2015. MetS was defined by the NCEP ATP III-2005 criterion. Complex survey design factors including sample weights, clustering, and stratification were considered for all analyses with instructions for using NHANES data. Cox proportional hazards models were used to estimate hazard ratios (HRs) and 95% CIs for mortality from all causes, heart disease, diabetes, and cancer.

**Results:**

We observed 8494 deaths during the 16.71 years of follow-up. Compared with those without MetS, individuals with MetS were associated with a significantly elevated multiadjusted HR of 1.24 (95% CI 1.16-1.33), 1.44 (95% CI 1.25-1.66), and 5.15 (95% CI 3.15-8.43) for all cause, heart diseases, and diabetes mellitus, respectively, whereas no significant association was found for cancer mortality (HR 1.17, 95% CI 0.95-1.43).

**Conclusions:**

Our study provides additional evidence that MetS and its components are significantly associated with all-cause, heart disease, and diabetes mortality, but not with cancer mortality. Health care professionals should pay more attention to MetS and its individual component.

## Introduction

Metabolic syndrome (MetS) is a common metabolic disorder that results from the increasing prevalence of obesity [1], which has been an increasing concern in the past few years [2]. A large number of studies have been conducted to explore the definition, prevalence, and associated factors of MetS [3-5], as well as to examine the relationship between MetS and cardiovascular disease (CVD) [6], diabetes mellitus (DM) [7], and other disease such as skin symptoms [8,9]. MetS is highly prevalent in many countries such as the United States [10], China [2], and India [11], despite the definitions of MetS being varied. MetS is a well-known, health-related problem.

Previous evidence indicated that MetS was associated with mortality; however, different definitions of MetS were used. One study [12] was conducted from 1984 to 1989 among men aged 42-60 years using the World Health Organization (WHO) and modified National Cholesterol Education Program (NCEP) definitions for MetS; it consistently showed that CVD and all-cause mortality increased in 1209 men with MetS. Another study [13] indicated that the WHO definition of MetS identified adults with increased CVD morbidity and mortality, but the study was conducted among middle-aged adults about 30 years ago in Europe with a small sample size. A large-sample study [14] conducted in 1978 and 1987 examined the relationship between syndrome X and mortality; however, syndrome X was the initial concept of MetS, which was different from MetS. Besides, similar to the 2 studies above, the deaths were also coded according to the International Classification of Diseases (ICD), Ninth Revision. It is well known that MetS is becoming prevalent among young adults due to great changes in lifestyle, diet, and socioeconomic environment, and the definition of MetS has been updated or modified several times and changed drastically. In 2005, the Third Report of the US NCEP Expert Panel on Detection, Evaluation, and Treatment of High Blood Cholesterol in Adults (Adult Treatment Panel [ATP] III) updated again the definition of MetS according to the modified American Diabetes Association criteria for impaired fasting glucose, which has since been widely adopted in the United States and elsewhere due to its simplicity to use in a clinical setting and its advantage of avoiding emphasis on a single cause [15].

Therefore, it is necessary to conduct a novel study among other population and countries with a large sample size using the updated and widely used definition of MetS and death code to examine the association of MetS with all-cause and cause-specific mortality.

## Methods

### Study Design and Participants

The study population were obtained from the National Health and Nutrition Examination Survey (NHANES) III (1988-1994) and the continuous NHANES (1999-2014), and data were obtained by questionnaire and interview, mobile physical examination, and laboratory tests with a complex, multistage, and probability sampling method. Details of NHANES have been described on the web [16]. In this analysis, 33,994 participants from the NHANES III (1988-1994) and 82,091 from the continuous NHANES (1999-2014) data sets were first enrolled. After excluding those without high-density lipoprotein cholesterol (HDL-C), blood pressure, plasma fasting glucose, triglycerides (TGs), and waist circumference data, 15,530 participants from the NHANES III and 25,371 from the continuous NHANES remained. Furthermore, we excluded participants aged <18 years and those without mortality data. Finally, a total of 36,414 participants were retained in our cohort for analysis. The flowchart of the study is presented in Figure S1 in Multimedia Appendix 1.

### Ascertainment of MetS

MetS was defined by the NCEP ATP III-2005 criterion [15], that is, a person who has 3 or more of the following criteria: (1) elevated waist circumference (EWC): waist circumference ≥102 cm in men and ≥88 cm in women; (2) elevated blood pressure: blood pressure ≥130/85 mm Hg or drug treatment of previously diagnosed hypertension; (3) reduced HDL-C: <40 mg/dL in men and <50 mg/dL in women or specific treatment for reduced HDL-C; (4) elevated TGs: TG level ≥150 mg/dL or drug treatment for elevated TG; and (5) elevated fasting glucose: fasting glucose level of ≥100 mg/L or drug treatment for elevated glucose and previously diagnosed type 2 diabetes.

### Ascertainment of Death

Mortality status was ascertained by probabilistic matching to the National Death Index through December 31, 2015, using a unique study identifier. Details of the matching method are available from the National Center for Health Statistics [17]. Causes of deaths were classified according to the codes of ICD-10. Primary outcomes in this study were mortality from all causes, heart diseases (codes I00-I09, I11, I13, and I20-I51), cancer (codes C00-C97), and diabetes (codes E10-E14).

### Ascertainment of Covariates

Gender, age, race and ethnicity (Mexican American, non-Hispanic Black, non-Hispanic White, or others), educational level (less than high school, high school or equivalent, or college or above), marital status (married; separated, including widowed and divorced groups; or never married), smoking, physical activity, and BMI were obtained by interviews and physical examinations. BMI was calculated as weight (kg) divided by height squared (m^2^) and was categorized into 3 groups: <25, 25-30, and ≥30 kg/m^2^ [18]. Family poverty-to-income ratio (PIR) level was grouped into 3 categories: 0-1.0, 1.1-3.0, and >3.0 [19]. Current smokers were defined as those who smoked at least 100 cigarettes in their lifetime and smoked at the time of survey. Baseline moderate-to-vigorous leisure physical activity level was defined as active physical activity such as brisk walking, carrying or lifting light or heavy loads, running, basketball, bicycling, swimming, or volleyball for at least 10 minutes continuously. Self-reported general health condition was classified 3 groups: very good to excellent, good, and poor to fair. Multiple imputation was used for missing values [20].

### Statistical Analysis

Complex survey design factors including sample weights, clustering, and stratification were considered for all analyses with instructions for using NHANES data. We compared baseline characteristics by MetS in the 2 intervals by using the Rao-Scott chi-square test for categorical variables and ANOVA and the Kruskal-Wallis test adjusted for sampling weights for continuous variables. The Kaplan-Meier method was used to plot the survival curves associated with MetS and the number of MetS components. Cox proportional hazards models were used to estimate the hazard ratios (HRs) with 95% CIs for all-cause and cause-specific morality for MetS and its components.

The baseline age (years, continuous), gender, and race and ethnicity (Mexican American, non-Hispanic Black, non-Hispanic White, or others) were adjusted in model 2. Furthermore, education level (less than high school, high school or equivalent, or college or above), family PIR level (0-1.0, 1.1-3.0, or >3.0), marital status (married, separated, or never married), BMI (<25, 25-30, or ≥30 kg/m^2^), smoking status (yes or no), active physical activity level (yes or no), self-reported health status (very good to excellent, good, or poor to fair) were adjusted in model 3. For analyses of one of the components of MetS and mortality, we further adjusted the other 4 components in model 4. We also conducted stratified analyses according to each covariate. We conducted several sensitivity analyses to test the robustness of the results. First, we removed participants with missing values for covariates and ran complete case analyses. Second, we excluded individuals with prevalent CVD, cancer, or DM to minimize potential reverse causation due to severe illness. Third, we excluded individuals who had a follow-up time of less than 3 years (including those who died within 3 years of follow-up).

All statistical analyses were performed using SAS (version 9.4; SAS Institute Inc) and R (version 4.0.5; R Foundation for Statistical Computing), with 2-sided *P*<.05 considered statistically significant.

### Ethical Considerations

This study proposal was approved by the Ethics and Human Subject Committee of Tongji Medical College, Huazhong University of Science and Technology (2019 IEC (S342)).

Written informed consent was obtained from all study participants in the NHANES, and the NHANES was approved by the Ethics Review Board of the National Centers for Health Statistics [21]. The NHANES data are free for public use and available on the web [22].

## Results

Baseline characteristic of the population are shown in Table 1. The median follow-up for survivors was 16.71 (95% CI 15.17-18.25) years. Among the 36,414 individuals, the weighted mean age was 48.07 (range 18-90) years: 57.22 (SD 16.637) years for those with MetS and 42.18 (SD 18.496) years for those without MetS. A total of 18,887 (51.87%) were women, 14,109 (38.75%) were younger adults aged <40 years, and 17,079 (46.9%) had good or excellent general health. Among all the participants, 39.17% (n=14,265) had MetS. Significant differences were observed in all groups except for the group of gender (all *P*<.05).

**Figure 1:**
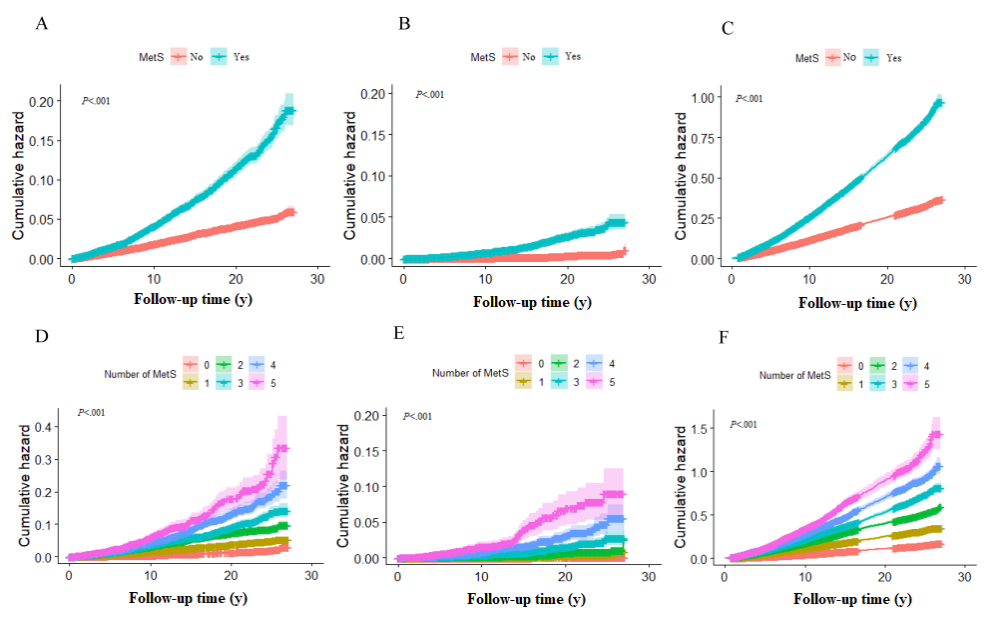
Unadjusted Kaplan-Meier hazard curves: (A) metabolic syndrome (MetS) and heart disease; (B) MetS and diabetes mellitus; (C) MetS and all causes; (D) the number of MetS components and heart disease; (E) the number of MetS components and diabetes mellitus; and (F) the number of MetS components and all causes.

There were 8494 deaths during the follow-up: 1428 (16.81%) deaths from heart diseases, 1280 (15.07%) deaths from cancer, and 220 (2.59%) deaths from DM. Table 2 demonstrates the associations of MetS and its components with all-cause and cause-specific mortality. Furthermore, we explored the relationships between the number of MetS components and mortality; similar findings were observed, and significant positive relationships were found between the different number of MetS components and all-cause, heart disease, and DM mortality, not for cancer mortality. The hazard of mortality increases with an increase in the number of MetS components. Besides, except for elevated TG, the other 4 components were associated with an increased hazard of all-cause mortality after adjusting for all covariates. Elevated blood pressure and EWC were not associated with heart disease mortality, whereas elevated TG and EWC were not associated with DM mortality. Interestingly, reduced HDL-C was associated with the hazard of cancer mortality (HR 1.36, 95% CI 1.12-1.64). Furthermore, the results of the sensitivity analyses did not change substantially (Tables S1-3 in Multimedia Appendix 1). In addition, Figure 1 shows that the cumulative hazard gradually increased with MetS and the number of MetS components during the follow-up time (all *P*<.001), and cumulative hazard was higher among adults with MetS and an increased number of MetS components (*P*<.001).

The subgroup analyses of MetS with all-cause, heart disease, and DM mortality are showed in Table 3. In the stratified analyses, significant associations between MetS and all-cause mortality were found among most groups, especially for groups of marital status and education levels. The relationships only existed for the separated marital status and did not exist among those with high school or equivalent educational levels. Similar findings were observed in the associations of MetS with heart disease and DM mortality. In addition, the positive relationship between MetS and heart disease mortality was found among non-Hispanic White individuals (HR 1.21 95% CI 1.11-1.32), those with PIR >3.0 (HR 1.82; 95% CI 1.46-2.28), and those with active physical activity (HR 1.53, 95% CI 1.30-1.81). Besides, individuals aged <40 years and those with PIR from 0 to 1.0 were not associated with elevated hazard of DM mortality. Men with MetS had higher hazard of heart disease mortality (1.54, 95% CI 1.23-1.93 for men; 1.38, 95% CI 1.16-1.65 for women), whereas women with MetS had higher hazard of DM mortality (8.75, 95% CI 4.20-18.21 for women; 3.60, 95% CI 1.63-7.98 for men).

**Table 1:** Baseline characteristics of participants from the National Health and Nutrition Examination Survey (NHANES) according to metabolic syndrome (MetS)^a^.

Characteristic			All participants (n=36,414)		MetS (n=14,265, 39.17%)		Non-MetS (n=22,149, 60.83%)		Rao-Scott *χ*^2^ (*df*)		*P* value
**Gender, n (%)**									5.55 (1)		.02
	Man	17,527 (48.13)		6746 (47.29)		10,781 (48.67)					
	Woman	18,887 (51.87)		7519 (52.17)		11,368 (51.33)					
**Age (years), mean (SD)**			48.07 (19.25)		57.22 (16.64)		42.18 (18.50)		1798.875 (2)		<.001
	<40, n (%)	14,109 (38.75)		2440 (17.1)		11,669 (52.68)					
	40-60, n (%)	10,409 (28.59)		4542 (31.84)		5867 (26.49)					
	≥60, n (%)	11,896 (32.67)		7283 (51.06)		4613 (20.83)					
**Race and ethnicity, n (%)**									12.94 (3)		.005
	Mexican American	8279 (22.74)		3259 (22.85)		5020 (22.66)					
	Non-Hispanic Black	8446 (23.19)		2905 (20.36)		5541 (25.02)					
	Non-Hispanic White	16,036 (44.04)		6734 (47.21)		9302 (42)					
	Others	3653 (10.03)		1367 (9.58)		2286 (10.32)					
**Educational levels, n (%)**									64.47 (2)		<.001
	Less than high school	17,243 (47.35)		7208 (50.53)		10,035 (45.31)					
	High school or equivalent	7053 (19.45)		2778 (19.47)		4305 (19.44)					
	College or above	12,088 (33.2)		4279 (30)		7809 (35.26)					
**Married status, n (%)**									314.18 (2)		<.001
	Married	11,193 (30.74)		5086 (35.65)		6107 (27.57)					
	Separated	16,809 (46.16)		7345 (51.49)		9464 (42.73)					
	Never married	8412 (23.1)		1834 (12.86)		6578 (29.7)					
**Family poverty-to-income ratio level, n (%)**									32.39 (2)		<.001
	0-1.0	7420 (20.38)		2934 (20.57)		4486 (20.25)					
	1.1-3.0	15,846 (43.52)		6435 (45.11)		9411 (42.49)					
	>3.0	13,148 (36.11)		4896 (34.32)		8252 (37.26)					
**BMI (kg/m^2^), n (%)**									4642.97 (2)		<.001
	<25	12,750 (35.01)		1697 (11.9)		11,053 (49.9)					
	25-30	12,441 (34.17)		5072 (35.56)		7369 (33.27)					
	≥30	11,223 (30.82)		7496 (52.55)		3727 (16.83)					
**Smoking status, n (%)**									22.59 (1)		<.001
	Yes	17,606 (48.35)		7353 (51.55)		10,253 (46.29)					
	No	18,808 (51.65)		6912 (48.45)		11,896 (53.71)					
**Active physical activity, n (%)**									190.66 (1)		<.001
	Yes	15,726 (43.19)		4858 (34.06)		10,868 (49.07)					
	No	20,688 (56.81)		9407 (65.94)		11,281 (50.93)					
**General health condition, n (%)**									938.15 (2)		<.001
	Very good to excellent	17,079 (46.9)		4523 (31.71)		12,556 (56.69)					
	Good	13,292 (36.5)		6052 (42.43)		7240 (32.69)					
	Poor to fair	6043 (16.60)		3690 (25.87)		2353 (10.62)					
Follow-up time (years), median (95% CI)			16.71 (15.17-18.25)		12.48 (11.93-13.03)		21.63 (21.30-21.97)		990.1294^b^		<.001
**Self-reported chronic diseases (including CVD^c^, cancer, and DM^d^), n (%)**									2083.75 (1)		<.001
	Yes	8324 (22.86)		5677 (39.8)		2647 (11.95)					
	No	28,090 (77.14)		8588 (60.2)		19,502 (88.05)					

^a^All estimates accounted for complex survey designs, and *P* values were calculated using ANOVA adjusting for sampling weights and Rao-Scott chi-square test for continuous and categorical variables.

^b^Kruskal-Wallis test was used.

^c^CVD: cardiovascular disease.

^d^DM: diabetes mellitus.

**Table 2:** Associations of metabolic syndrome (MetS) and its components and all-cause and specific cause mortality.

Association			Hazard ratio (95% CI)						
			All causes (n=8494)		Heart disease (n=1428)		Cancer (n=1280)		DM^a^ (n=220)
**MetS**									
	Model 1^b^	2.90 (2.66-3.16)		3.56 (3.16-4.01)		2.35 (1.97-2.80)		12.19 (7.48-19.84)	
	Model 2^c^	1.30 (1.21-1.40)		1.49 (1.33-1.67)		1.49 (0.98-1.43)		6.34 (3.70-10.87)	
	Model 3^d^	1.24 (1.16-1.33)		1.44 (1.25-1.66)		1.17 (0.95-1.43)		5.15 (3.15-8.43)	
**Number of MetS^e^ (reference=0)**									
	1	1.29 (1.12-1.48)		1.31 (0.82-2.09)		1.04 (0.75-1.43)		11.65 (1.99-68.17)	
	2	1.41 (1.21-1.63)		1.70 (1.10-2.64)		1.11 (0.80-1.53)		10.76 (2.08-55.77)	
	3	1.51 (1.28-1.78)		1.82 (1.27-2.61)		1.26 (0.88-1.81)		29.53 (5.63-154.93)	
	4	1.70 (1.48-1.96)		2.51 (1.63-3.88)		1.20 (0.86-1.68)		56.62 (10.20-314.33)	
	5	2.01 (1.68-2.40)		2.77 (1.68-4.56)		1.32 (0.93-1.89)		138.08 (24.35-783.13)	
**Components of MetS^e^**									
	EBP^f^	1.15 (1.07-1.24)		1.15 (0.96-1.38)		0.91 (0.77-1.08)		2.23 (1.29-3.85)	
	EGLU^g^	1.18 (1.10-1.27)		1.38 (1.17-1.63)		1.03 (0.87-1.21)		6.66 (3.49-12.71)	
	ETG^h^	1.14 (1.09-1.21)		1.43 (1.24-1.65)		1.05 (0.89-1.25)		1.76 (1.01-3.08)	
	Reduced HDL-C^i^	1.24 (1.16-1.32)		1.39 (1.17-1.64)		1.35 (1.13-1.60)		3.05 (2.00-4.64)	
	EWC^j^	1.17 (1.08-1.27)		1.12 (0.87-1.43)		1.09 (0.89-1.34)		1.89 (1.17-3.06)	
**Components of MetS^k^**									
	EBP	1.12 (1.04-1.21)		1.09 (0.91-1.30)		0.91 (0.77-1.08)		1.79 (1.04-3.10)	
	EGLU	1.14 (1.06-1.23)		1.32 (1.11-1.57)		1.01 (0.85-1.20)		5.77 (3.00-11.10)	
	ETG	1.05 (0.99-1.12)		1.29 (1.09-1.53)		0.96 (0.80-1.16)		1.07 (0.61-1.89)	
	Reduced HDL-C	1.20 (1.11-1.29)		1.25 (1.04-1.50)		1.36 (1.12-1.64)		2.56 (1.63-4.03)	
	EWC	1.12 (1.03-1.22)		1.04 (0.81-1.32)		1.07 (0.88-1.31)		1.42 (0.90-2.23)	

^a^DM: diabetes mellitus.

^b^Model 1: unadjusted.

^c^Model 2: adjusted for age (continuous), gender, and race.

^d^Model 3: model 2 + educational levels, marriage status, family poverty-to-income ratio level, BMI (category), smoking, physical activity, and general health condition.

^e^Model 3.

^f^EBP: elevated blood pressure.

^g^EGLU: elevated fasting glucose.

^h^ETG: elevated triglycerides.

^i^HDL-C: high-density lipoprotein cholesterol.

^j^EWC: elevated waist circumference.

^k^Model 3+adjusted for other 4 components.

**Table 3:** Stratified analyses on associations of metabolic syndrome (MetS) and its components and all-cause and specific cause mortality (adjusted for other covariates).

Characteristic		Hazard ratio (95% CI)		
		All causes	Heart disease	DM^a^
**Gender**				
	Man	1.23 (1.13-1.34)	1.54 (1.23-1.93)	3.60 (1.63-7.98)
	Woman	1.26 (1.14-1.39)	1.38 (1.16-1.65)	8.75 (4.20-18.21)
**Age group (years)**				
	<40	2.02 (1.49-2.74)	3.55 (1.01-12.46)	0.69 (0.18-2.63)
	40-60	1.43 (1.18-1.73)	1.67 (1.06-2.63)	9.99 (3.87-25.80)
	≥60	1.17 (1.08-1.27)	1.33 (1.15-1.54)	4.55 (2.46-8.43)
**Race and ethnicity**				
	Mexican American	1.20 (1.07-1.34)	1.27 (0.83-1.93)	2.89 (1.30-6.45)
	Non-Hispanic Black	1.34 (1.18-1.52)	1.25 (0.91-1.71)	4.62 (2.59-8.26)
	Non-Hispanic White	1.21 (1.11-1.32)	1.45 (1.22-1.73)	5.54 (2.78-11.02)
	Others	1.59 (1.07-2.36)	2.30 (0.67-7.93)	N/A^b^
**Educational levels**				
	Less than high school	1.26 (1.18-1.36)	1.39 (1.17-1.65)	4.94 (3.11-7.84)
	High school or equivalent	1.19 (0.98-1.44)	1.07 (0.51-2.24)	1.00 (0.28-3.52)
	College or above	1.18 (1.01-1.38)	1.99 (1.27-3.10)	31.27 (8.22-118.85)
**Married status**				
	Married	1.18 (0.92-1.50)	1.57 (0.25-9.97)	0.93 (0.19-4.54)
	Separated	1.23 (1.15-1.32)	1.40 (1.20-1.64)	5.42 (3.17-9.30)
	Never married	1.00 (0.67-1.50)	1.44 (0.60-3.47)	9.22 (0.53-162.16)
**Family poverty-to-income ratio level**				
	0-1.0	1.30 (1.07-1.57)	1.19 (0.74-1.90)	1.61 (0.67-3.87)
	1.1-3.0	1.12 (1.03-1.23)	1.26 (0.99-1.60)	8.38 (3.00-23.46)
	>3.0	1.36 (1.21-1.53)	1.82 (1.46-2.28)	6.03 (2.96-12.29)
**BMI (kg/m^2^)**				
	<25	1.22 (1.11-1.35)	1.40 (1.07-1.83)	5.57 (2.65-11.73)
	25-30	1.14 (1.02-1.27)	1.33 (1.06-1.68)	2.91 (1.35-6.27)
	≥30	1.45 (1.18-1.77)	1.72 (1.11-2.66)	13.83 (4.58-41.76)
**Smoking status**				
	Yes	1.28 (1.15-1.42)	1.58 (1.25-1.99)	5.60 (3.01-10.44)
	No	1.18 (1.08-1.30)	1.30 (1.08-1.57)	4.65 (1.92-11.29)
**Active physical activity**				
	Yes	1.28 (1.15-1.42)	1.53 (1.30-1.81)	4.17 (2.12-8.21)
	No	1.17 (1.05-1.31)	1.27 (0.92-1.75)	7.71 (3.32-17.91)
**General health condition**				
	Very good to excellent	1.25 (1.12-1.40)	1.76 (1.37-2.25)	3.58 (1.72-7.45)
	Good	1.26 (1.13-1.40)	1.24 (1.02-1.51)	8.94 (2.60-30.73)
	Poor to fair	1.14 (1.01-1.28)	1.44 (1.10-1.89)	5.23 (1.84-14.82)

^a^DM: diabetes mellitus.

^b^N/A: not applicable.

## Discussion

### Principal Findings

Our study suggested that MetS was associated with a significantly elevated hazard of all-cause, heart disease, and DM mortality, and the associations were also significant after adjusting for sociodemographic factors, lifestyle factors, and health status. Existing studies have reported consistent results regarding the relationship between MetS and the risk of CVD mortality [13], in which MetS was defined using the WHO definition, and our study provided further evidence of the association between MetS and mortality hazard, using the NCEP ATP Ⅲ definition. However, the nonsignificant association of MetS and all-cause mortality was observed in another study of men with the NCEP ATP III definition (relative risk 1.67, 95% CI 0.91-3.08) [12], and our study showed that men with MetS had an increased hazard of all-cause mortality. Different definitions of MetS may be a main cause. In addition, population differences and sample size may also be the important factors. Moreover, the previous study only included 1029 men aged 42-60 years. Our study was conducted with a large sample size among adults aged 18-90 years, which could provide more information. The findings may have important public health implications. MetS is prevalent in many areas around the world and has been reported to be associated with several diseases. Our study provides supportive evidence on a positive association between MetS and mortality, which indicates an urgent need of the prevention and control of MetS.

Previous studies have revealed that the number of MetS components was related to DM and CVD in a dose-response way [6,7], which may explain why individuals with more MetS components had higher hazards of all-cause, heart disease, and DM mortality in this study. Furthermore, there were great differences between the components of MetS and mortality, especially for heart disease and DM mortality, which may be due to the different pathological mechanisms and the interactive effects of the components [23-25]. In addition, our study suggested that MetS was not associated with cancer mortality, but a reduced HDL-C level may increase the hazard of cancer mortality. Previous studies showed that metabolic abnormalities or the underlying insulin resistance; specific cancer sites such as breast and uterine cancer [26,27]; and cancer mortality risk tended to increase with an increasing number of metabolic abnormalities in women but not in men [14]. More epidemiological studies and pathological mechanisms on the issue should be conducted and discussed.

Furthermore, our study indicated that there were significant associations of MetS with all-cause, heart disease, and DM mortality among individuals with the separated marital status, including the widowed and divorced groups, but not among those with other marital statuses. This suggests that health care professionals need pay more attention to these individuals. Besides, we also found significant associations of MetS with heart disease and DM mortality among the population with high PIR and the non-Hispanic White population. High prevalence of unhealthy lifestyle and risk factors of cardiometabolic diseases in high-income populations [28] and the fact that obesity prevalence varied by racial and ethnic group [29] may result in this phenomenon. In addition, men with MetS had higher risk of heart disease mortality, whereas women with MetS had higher risk of DM mortality. Gender differences in various disease and related risk factors has been observed [30,31], which may be due to the biology of gender; however, variation in cultural, societal, and historical contexts could also result in different life experiences of men and women and variation in the mortality [32]. Therefore, public health policies should recognize variations across genders as well as incorporate cultural and societal factors within and across countries [33]. Besides, a higher HRs was observed in all-cause, heart disease, and DM mortality among smoking individuals, which is easy to understand considering the obvious and well-known hazards of smoking to human health [34,35]. Our study once again suggested that intervention in smokers may contribute to reducing mortality.

This study had several strengths. First, the prospective cohort was well designed with a large and nationally representative sample and a reliable assessment of cause of deaths. Second, we conducted detailed analyses of MetS and its components and mortality, and we stratified the analyses. Besides, we adjusted for a large number of potential confounders, including socioeconomic status and lifestyle factors. Some limitations should also be acknowledged. First, the limited number of DM deaths may affect the validity; however, it does not seem to have a significant influence on the main findings of the study. Second, many covariates were mainly self-reported and were available only at baseline. Thus, it was impossible to use time-varying covariates to capture changes in the possible confounders over time. Third, our findings may only be representative of US residents, and further study should be conducted to validate the generalizability to other populations. Fourth, people with diabetes often die from complications of diabetes, such as heart and kidney diseases, and the cause of death may be reported as heart disease, not diabetes. Besides, lifestyle changes might have occurred since the survey, which may affect the results. Furthermore, people without a diagnosis of diabetes, with parameters in the normal or prediabetes range, may progress to type 2 diabetes along with its complications, which also might influence the results. Last, but not least, due to the nature of observational studies, residual confounding was still possible.

### Conclusion

In conclusion, MetS is associated with higher hazard of all-cause, heart disease, and DM mortality among US adults. Future studies are needed to reveal the mechanisms underlying the association between MetS and mortality. Both the identification of MetS using simple and efficient criteria and the early prevention and treatment are of great importance to improving the healthy life expectancy of a population, which should be of concern to health care professionals.

## Appendix

Multimedia Appendix 1 Sensitivity analyses, flowchart of the study, and supplementary methods.

## Abbreviations

ATP: Adult Treatment Panel
CVD: cardiovascular disease
DM: diabetes mellitus
EWC: elevated waist circumference
HDL-C: high-density lipoprotein cholesterol
HR: hazard ratio
ICD: International Classification of Diseases
MetS: metabolic syndrome
NCEP: National Cholesterol Education Program
NHANES: National Health and Nutrition Examination Survey
PIR: poverty-to-income ratio
TG: triglyceride
WHO: World Health Organization

## Data Availability

All the data used in this study are available on the web [36].
