# Supplementary material for: Association Between Metabolic Syndrome and Mortality: Prospective Cohort Study
Source: JMIR Public Health Surveill. 2023 Sep 5;9:e44073. doi: 10.2196/44073 (PMC10509744; doi:10.2196/44073)
Supplement: Multimedia Appendix 1 [file publichealth_v9i1e44073_app1.docx]

| **Table S1. Associations of MetS and its components and all-cause and specific cause mortality, sensitivity analyses^#^** | | | | |
| --- | --- | --- | --- | --- |
|  | **Hazard ratio (95% CI)** | | | |
|  | **Total** | **Heart Diseases** | **Cancer** | **DM** |
| MetS |  |  |  |  |
| Model 1 | 2.92 (2.66-3.20) | 3.63 (3.19-4.13) | 2.44 (2.02-2.96) | 12.22 (7.31-20.44) |
| Model 2 | 1.30 (1.20-1.41) | 1.50 (1.31-1.70) | 1.22 (0.99-1.50) | 6.44 (3.64-11.41) |
| Model 3 | 1.22 (1.13-1.32) | 1.40 (1.19-1.65) | 1.18 (0.93-1.49) | 4.57 (2.68-7.79) |
| ^*^Number of MetS (ref.=0) |  |  |  |  |
| 1 | 1.29 (1.10-1.52) | 1.50 (0.93-2.40) | 1.02 (0.72-1.45) | 11.15 (1.85-67.10) |
| 2 | 1.45 (1.21-1.73) | 1.99 (1.28-3.08) | 1.15 (0.81-1.64) | 9.61 (1.79-51.69) |
| 3 | 1.50 (1.24-1.81) | 2.03 (1.41-2.93) | 1.27 (0.86-1.89) | 24.73 (4.48-136.35) |
| 4 | 1.73 (1.46-2.05) | 2.83 (1.84-4.36) | 1.29 (0.90-1.86) | 46.90 (7.56-290.81) |
| 5 | 2.04 (1.65-2.53) | 3.03 (1.80-5.10) | 1.33 (0.85-2.06) | 112.14 (17.97-699.74) |
| ^*^Components of MetS |  |  |  |  |
| EBP | 1.15 (1.05-1.26) | 1.18 (0.99-1.40) | 0.91 (0.76-1.10) | 1.99 (1.15-3.47) |
| EGLU | 1.20 (1.11-1.30) | 1.44 (1.22-1.70) | 1.06 (0.89-1.26) | 5.85 (3.02-11.33) |
| ETG | 1.16 (1.09-1.23) | 1.43 (1.22-1.68) | 1.06 (0.88-1.28) | 1.71 (0.92-3.16) |
| Reduced HDL-C | 1.23 (1.15-1.32) | 1.33 (1.10-1.60) | 1.37 (1.12-1.67) | 3.02 (1.86-4.92) |
| EWC | 1.11 (1.01-1.23) | 1.03 (0.78-1.35) | 1.08 (0.86-1.35) | 1.68 (0.93-3.03) |
| ^**^Components of MetS |  |  |  |  |
| EBP | 1.12 (1.03-1.23) | 1.11 (0.94-1.32) | 0.91 (0.76-1.09) | 1.63 (0.93-2.87) |
| EGLU | 1.17 (1.08-1.27) | 1.39 (1.16-1.65) | 1.05 (0.87-1.25) | 5.16 (2.62-10.13) |
| ETG | 1.07 (0.99-1.14) | 1.31 (1.09-1.57) | 0.96 (0.78-1.18) | 1.07 (0.58-1.98) |
| Reduced HDL-C | 1.19 (1.10-1.28) | 1.19 (0.97-1.46) | 1.38 (1.11-1.71) | 2.59 (1.55-4.30) |
| EWC | 1.07 (0.97-1.18) | 0.96 (0.73-1.26) | 1.05 (0.84-1.31) | 1.29 (0.73-2.27) |

**^#^**Excluded participants with missing values for covariates

MetS= metabolic syndrome; DM= diabetes mellitus; BMI=body mass index; EBP=elevated blood pressure; ETG= elevated triglycerides; EGLU= elevated fasting glucose; HDL-C= high-density lipoprotein cholesterol; EWC =elevated waist circumference;

Model 1: unadjusted;

Model 2: adjusted for age (continuous), gender, race;

Model 3: model 2 + educational levels, marriage status, family income-poverty ratio level, BMI (category), smoking, physical activity, general health condition

*Model 3

**Model 3+adjusted other four components

| **Table S2. Associations of MetS and its components and all-cause and specific cause mortality, sensitivity analyses^#^** | | | | |
| --- | --- | --- | --- | --- |
|  | **Hazard ratio (95% CI)** | | | |
|  | **Total** | **Heart Diseases** | **Cancer** | **DM** |
| MetS |  |  |  |  |
| Model 1 | 2.70 (2.42-3.01) | 3.43 (2.79-4.22) | —— | 18.97 (7.22-49.83) |
| Model 2 | 1.22 (1.10-1.35) | 1.41 (1.15-1.74) | 1.24 (0.97-1.59) | 10.26 (4.09-25.75) |
| Model 3 | 1.16 (1.04-1.29) | 1.29 (1.01-1.66) | 1.18 (0.90-1.54) | 5.91 (2.18-16.06) |
| ^*^Number of MetS (ref.=0) |  |  |  |  |
| 1 | 1.31 (1.10-1.57) | 1.52 (0.84-2.78) | 1.16 (0.75-1.80) | 3.23 (0.42-25.06) |
| 2 | 1.43 (1.17-1.74) | 1.82 (1.01-3.28) | 1.26 (0.85-1.88) | 1.43 (0.19-10.57) |
| 3 | 1.45 (1.17-1.81) | 1.78 (1.08-2.94) | 1.34 (0.88-2.04) | 10.81 (1.71-68.41) |
| 4 | 1.67 (1.38-2.03) | 2.60 (1.58-4.29) | 1.51 (1.00-2.29) | 12.97 (1.83-91.79) |
| 5 | 1.68 (1.28-2.21) | 2.33 (1.15-4.72) | 1.50 (0.87-2.59) | 11.55 (1.12-119.50) |
| ^*^Components of MetS |  |  |  |  |
| EBP | 1.19 (1.08-1.32) | 1.14 (0.89-1.47) | 0.99 (0.81-1.20) | 1.51 (0.49-4.64) |
| EGLU | 1.07 (0.96-1.19) | 1.21 (0.95-1.55) | 1.01 (0.81-127) | 2.95 (1.07-8.18) |
| ETG | 1.10 (1.00-1.21) | 1.32 (1.05-1.66) | 1.11 (0.89-1.39) | 0.82 (0.29-2.31) |
| Reduced HDL-C | 1.20 (1.11-1.30) | 1.44 (1.15-1.80) | 1.36 (1.08-1.71) | 3.84 (1.65-8.93) |
| EWC | 1.10 (0.94-1.29) | 1.00 (0.71-1.40) | 1.17 (0.86-1.58) | 1.67 (0.62-4.74) |
| ^**^Components of MetS |  |  |  |  |
| EBP | 1.18 (1.07-1.30) | 1.10 (0.86-1.41) | 0.98 (0.81-1.19) | 1.42 (0.44-4.58) |
| EGLU | 1.05 (0.94-1.17) | 1.18 (0.91-1.51) | 0.99 (0.79-1.25) | 2.64 (0.90-7.80) |
| ETG | 1.01 (0.91-1.13) | 1.17 (0.91-1.52) | 1.01 (0.78-1.31) | 0.48 (0.16-1.48) |
| Reduced HDL-C | 1.19 (1.09-1.30) | 1.36 (1.06-1.74) | 1.35 (1.03-1.76) | 4.32 (1.59-11.71) |
| EWC | 1.06 (0.91-1.25) | 0.93 (0.67-1.30) | 1.13 (0.84-1.52) | 1.46 (0.57-3.75) |

**^#^E**xcluded participants with prevalent diabetes, cardiovascular disease, or cancer

MetS= metabolic syndrome; DM= diabetes mellitus; BMI=body mass index; EBP=elevated blood pressure; ETG= elevated triglycerides; EGLU= elevated fasting glucose; HDL-C= high-density lipoprotein cholesterol; EWC =elevated waist circumference;

Model 1: unadjusted;

Model 2: adjusted for age (continuous), gender, race;

Model 3: model 2 + educational levels, marriage status, family income-poverty ratio level, BMI (category), smoking, physical activity, general health condition

*Model 3

**Model 3+adjusted other four components

| **Table S3. Associations of MetS and its components and all-cause and specific cause mortality, sensitivity analyses^#^** | | | | |
| --- | --- | --- | --- | --- |
|  | **Hazard ratio (95% CI)** | | | |
|  | **Total** | **Heart Diseases** | **Cancer** | **DM** |
| MetS |  |  |  |  |
| Model 1 | 2.97 (2.70-3.26) | 3.63 (3.14-4.20) | 2.43 (1.98-2.99) | 11.77 (6.96-19.89) |
| Model 2 | 1.31 (1.20-1.43) | 1.49 (1.28-1.72) | 1.24 (0.99-1.55) | 6.08 (3.41-10.85) |
| Model 3 | 1.23 (1.13-1.34) | 1.39 (1.16-1.67) | 1.17 (0.91-1.50) | 4.06 (2.46-6.68) |
| ^*^Number of MetS (ref.=0) |  |  |  |  |
| 1 | 1.32 (1.11-1.57) | 1.59 (0.97-2.60) | 1.04 (0.72-1.51) | 10.58 (1.74-64.29) |
| 2 | 1.46 (1.20-1.78) | 2.13 (1.34-3.37) | 1.12 (0.77-1.64) | 8.43 (1.58-44.87) |
| 3 | 1.52 (1.23-1.88) | 2.13 (1.38-3.28) | 1.24 (0.80-1.92) | 21.09 (3.87-114.84) |
| 4 | 1.77 (1.48-2.11) | 3.03 (1.93-4.77) | 1.26 (0.85-1.88) | 35.09 (6.01-204.71) |
| 5 | 2.13 (1.71-2.66) | 3.20 (1.86-5.51) | 1.42 (0.89-2.27) | 91.48 (15.18-551.14) |
| ^*^Components of MetS |  |  |  |  |
| EBP | 1.18 (1.07-1.31) | 1.20 (1.01-1.42) | 0.94 (0.77-1.14) | 1.86 (1.08-3.19) |
| EGLU | 1.20 (1.11-1.30) | 1.41 (1.15-1.73) | 1.05 (0.86-1.28) | 5.56 (2.89-10.67) |
| ETG | 1.17 (1.09-1.26) | 1.44 (1.21-1.72) | 1.09 (0.89-1.33) | 1.59 (0.86-2.94) |
| Reduced HDL-C | 1.22 (1.14-1.31) | 1.35 (1.12-1.62) | 1.32 (1.06-1.65) | 2.79 (1.71-4.56) |
| EWC | 1.12 (1.01-1.24) | 1.03 (0.77-1.37) | 1.09 (0.86-1.39) | 1.71 (0.91-3.19) |
| ^**^Components of MetS |  |  |  |  |
| EBP | 1.16 (1.05-1.27) | 1.13 (0.96-1.34) | 0.93 (0.77-1.13) | 1.54 (0.88-2.69) |
| EGLU | 1.17 (1.08-1.27) | 1.36 (1.10-1.68) | 1.03 (0.84-1.27) | 4.94 (2.53-9.65) |
| ETG | 1.08 (1.00-1.17) | 1.32 (1.08-1.61) | 1.00 (0.81-1.24) | 1.01 (0.54-1.89) |
| Reduced HDL-C | 1.17 (1.08-1.27) | 1.21 (0.99-1.48) | 1.32 (1.04-1.67) | 2.41 (1.43-4.06) |
| EWC | 1.07 (0.97-1.19) | 0.96 (1.73-1.27) | 1.06 (0.84-1.35) | 1.36 (0.75-2.45) |

**^#^**Excluded participants who had follow-up time of less than three years (including those who died within three years of follow-up)

MetS= metabolic syndrome; DM= diabetes mellitus; BMI=body mass index; EBP=elevated blood pressure; ETG= elevated triglycerides; EGLU= elevated fasting glucose; HDL-C= high-density lipoprotein cholesterol; EWC =elevated waist circumference;

Model 1: unadjusted;

Model 2: adjusted for age (continuous), gender, race;

Model 3: model 2 + educational levels, marriage status, family income-poverty ratio level, BMI (category), smoking, physical activity, general health condition

*Model 3

**Model 3+adjusted other four components

**Figure S1.** Flowchart of the study


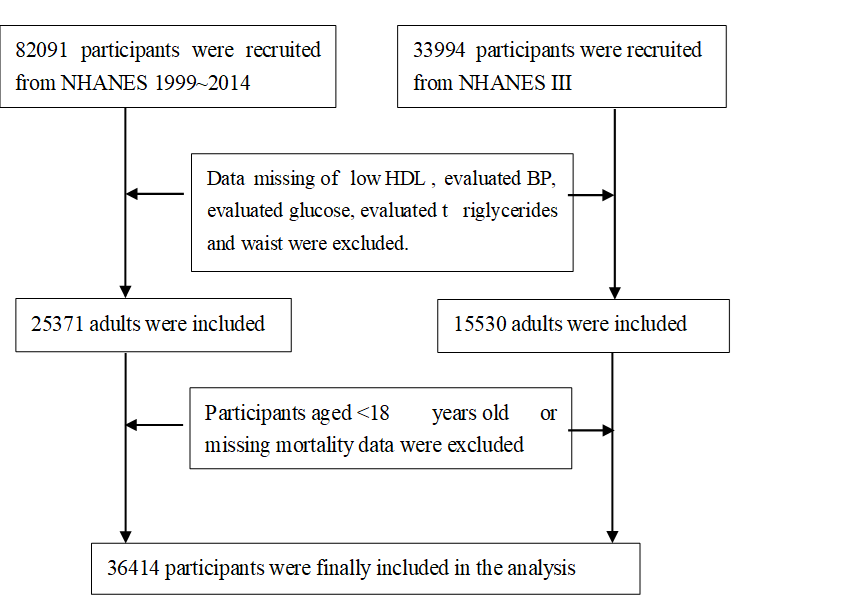


**Methods**

**Sample size calculation**

According to the formula for calculating the sample size of the cohort study, it is assumed that the number of people in the metabolic syndrome group and the nonmetabolic syndrome group is the same:


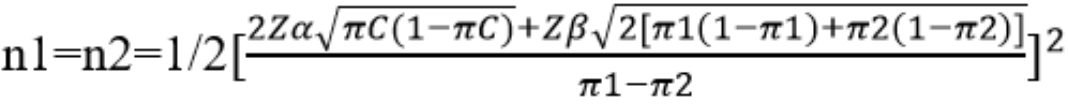
, we take both sides ɑ= 0.05, ß=0.10 based on previous data, assuming π1=0.20, π2=0.10, π c=0.15, and thus n1=n2=329. Considering the possible loss of follow-up, the sample size is 724 calculated based on a 10% loss of follow-up.

**Four models**

The baseline age (years, continuous), gender and race/ethnicity (non-Hispanic white, non-Hispanic black, Mexican American, and others) were adjusted in Model 2. Furthermore, education level (less than high school, high school or equivalent,

and college or above), family income-poverty ratio level (0-1.0, 1.1-3.0, >3.0), marital status (married, separated and never married), BMI (<25, 25~30 and ≥30 kg/ m2), smoking status (no, yes), active physical activity level (yes, no), self-reported health status (very good to excellent, good, and poor to fair) were adjusted in Model 3. For analyses of one of the components of MetS and mortality, we further adjusted the other four components in Model 4.

**Multiple imputation**

In longitudinal data, multiple imputation methods can be used for missing rates ranging from 10% to 40%. In the present study, the number of missing items varies for different variables. If all missing items are deleted, the total missing rate is 35.11%, thus, we conducted the multiple imputation, and we also conducted the sensitivity analysis excluded participants with missing values for covariates (Supplementary table 1). The imputed time =5, and Markov chain Monte Carlo (MCMC) was used in the present analysis.
